# Inadequate food intake among adults living with HIV

**DOI:** 10.1590/1516-3180.2013.1313478

**Published:** 2013-06-01

**Authors:** Kelly Virecoulon Giudici, Ana Clara Fonseca Leitão Duran, Patricia Constante Jaime

**Affiliations:** I BSc. Doctoral Student of Public Health Nutrition, Department of Nutrition, Faculdade de Saúde Pública, Universidade de São Paulo (FSP-USP), São Paulo, Brazil.; II PhD. Assistant Professor, Department of Nutrition, Faculdade de Saúde Pública, Universidade de São Paulo (FSP-USP), São Paulo, Brazil.

**Keywords:** Eating, Diet, HIV, Antiretroviral therapy, highly active, Adult, Ingestão de alimentos, Dieta, HIV, Terapia anti-retroviral de alta atividade, Adulto

## Abstract

**CONTEXT AND OBJECTIVE:**

The number of people living with HIV (PLHIV) in Brazil is between 600,000 and 890,000. Assessing the diet is important in planning healthcare actions and improving PLHIV’s quality of life. This study aimed to estimate the prevalence of inappropriate protein, total fat, saturated fat, carbohydrate, fiber, sodium, calcium and cholesterol intake among PVHIV on highly-active antiretroviral therapy (HAART).

**DESIGN AND SETTING:**

Cross-sectional study in nine Specialized STD/AIDS Healthcare Centers in São Paulo.

**METHODS:**

Men and women aged 20 to 59 years, on HAART for at least three months, were included. Nutrient intake was assessed using 24-hour food recall applied in person and repeated among 30% of the population by telephone. The between and within-person variances were corrected.

**RESULTS:**

507 individuals were evaluated: 58% male, mean age 41.7 years (standard deviation, SD = 7.8). The mean time since HIV diagnosis was 6.6 years (SD = 4.1), and since HAART onset, 5.1 years (SD = 3.3). More than 20% of the population presented intake above the recommendations for saturated fat, cholesterol and/or sodium, and below the recommendations for fiber. The recommended maximum tolerable sodium level was exceeded by 99% of the sample, and 86% of men and 94% of women did not reach the daily recommendations for calcium. Protein, carbohydrate and total fat intakes were adequate for the majority of the population.

**CONCLUSIONS:**

A significant portion of the population presented inappropriate intake of saturated fat, sodium, fiber and calcium. Interventions aimed at improving PLHIV’s dietary quality are needed.

## RESUMO

**CONTEXTO E OBJETIVO:**

O número de pessoas vivendo com HIV (PVH) no Brasil oscila entre 600.000 e 890.000. A avaliação de dietas é relevante para o planejamento de ações de saúde e melhoria da qualidade de vida desses indivíduos. Este estudo visa estimar a prevalência de inadequação do consumo de proteínas, lipídios totais, lipídios saturados, carboidratos, fibras, sódio, cálcio e colesterol em PVH em terapia antirretroviral de alta atividade (TARV).

**TIPO DE ESTUDO E LOCAL:**

Estudo transversal em nove Centros de Assistência Especializados em DST/Aids localizados em São Paulo.

**MÉTODOS:**

Homens e mulheres com idade entre 20 e 59 anos, em TARV por no mínimo 3 meses, foram incluídos. O consumo de nutrientes foi avaliado com um recordatório alimentar de 24 horas aplicado pessoalmente e repetido em 30% da população por telefone. A variabilidade intra e interpessoal foi corrigida.

**RESULTADOS:**

Foram avaliados 507 indivíduos, 58% homens e idade média de 41,7 anos (desvio padrão, DP = 7,8). O tempo médio de infecção por HIV foi de 6,6 anos (DP = 4,1), e de TARV, de 5,1 anos (DP = 3,3). Mais que 20% da população apresentou consumo acima das recomendações para lipídios saturados, colesterol e/ou sódio, e abaixo para fibras. Excederam a recomendação máxima tolerada para sódio 99% da amostra, enquanto 86% dos homens e 94% das mulheres não atingiram as recomendações diárias para o cálcio. O consumo de proteínas, carboidratos e lipídios totais mostrou-se adequado para a maioria da população.

**CONCLUSÕES:**

Uma importante parcela da população apresentou consumo inadequado para lipídios saturados, sódio, fibras e cálcio. Intervenções que visem melhorar a qualidade da dieta de PVH são necessárias.

## INTRODUCTION

Since the first notification of AIDS in Brazil, in 1980, the number of individuals infected by the human immunodeficiency virus (HIV) has been increasing greatly. Estimates of the numbers of people living with HIV (PLHIV) in Brazil range from 600,000 to 890,000 individuals,^1^ although the number of notified cases from 1980 to June 2011 total 608,230 (65.4% men), which corresponds to an incidence rate of 17.9 cases per 100,000 inhabitants.^2^

In 1996, universal access to highly-active antiretroviral therapy (HAART) through the public healthcare system was established by the Brazilian Ministry of Health. Meanwhile, the HIV-derived mortality rates have decreased, followed by a decline in associated opportunistic infections and hospitalization, along with an increase in survival.^2^ Fifteen years later, the Brazilian program of universal access to HAART benefits over 200,000 PLHIV,^3^ which corresponds to antiretroviral therapy coverage of 97% of live notified cases and 50 to 89% of the entire HIV-infected population in the country.^4^

Following these important changes, HIV infection has started to be considered a chronic and potentially manageable disease. However, side effects secondary to prolonged use of HAART have brought new demands for healthcare services specializing in caring for PLHIV.^5^^,^^6^

Among the side effects of HAART are gastrointestinal problems and lipodystrophy. The latter is a syndrome defined as a set of changes that includes fat loss in peripheral areas (face, buttocks, arms and legs) and fat gain in central portions of the body (abdomen, neck and chest). HAART also produces metabolic alterations as side effects, such as hypercholesterolemia, hypertriglyceridemia, insulin resistance and lactic acidosis.^7^^,^^8^ These metabolic changes do not affect all patients on HAART, and this supports the hypothesis that they are caused not only by this drug but also by genetics and behavioral variables like food consumption and stress.^8^

Healthier eating may offset or at least minimize these side effects, and this, along with the already known benefits of a healthy diet,^5^ gives rise to the need for food intake assessment in this group.

Assessing food intake has become important in planning actions relating to care for PLHIV, among the new demands that form part of caring for this population. Knowing how appropriate this group’s diet is plays an important role in planning healthcare actions, whether for monitoring or for intervening. Moreover, this provides backing for establishing hypotheses about the relationship between diet and health.^9^

## OBJECTIVE

This study aimed to investigate the prevalence of inappropriate intake of protein, total fat, saturated fat, carbohydrate, dietary fiber, sodium, calcium and cholesterol among people living with HIV who were on HAART in the city of São Paulo, Brazil.

## METHODS

Data on the participants in this cross-sectional study were gathered at nine of the fifteen Specialized Community Healthcare Centers for sexually transmitted diseases/acquired immunodeficiency syndrome (STD/AIDS) that are administered through the municipal STD/AIDS program. These Community Healthcare Centers were located in four different geographical regions of the city of São Paulo, and no socioeconomic differences were found when they were compared with the Centers that could not be included in the study due to infrastructure problems at the time of the data collection.

Adults (aged 20-59 years) living with HIV who had been on HAART for at least three months were invited to join the study. Participants were chosen using a consecutive cumulative approach between November 2007 and June 2008. Patients who agreed to participate were admitted after signing an informed consent form, and were fully informed about the objectives and procedures of the research.

After interviews, all the participants received brief nutritional counseling promoting healthy eating, physical activity and prevention of biochemical and morphological changes, in accordance with the recommendations of the Dietary Guidelines for the Brazilian Population^10^ and the Nutritional Recommendations for Individuals Living with HIV/AIDS.^11^ They were also referred to the dietitians at the STD/AIDS Specialized Community Healthcare Centers, if necessary. The Ethics Committees of both the School of Public Health, University of São Paulo (Faculdade de Saúde Pública, Universidade de São Paulo, FSP-USP) and the Health Department of the municipality of São Paulo approved this study. Subject anonymity and information confidentiality were ensured at all times.

Macronutrient intake (protein, total fat, saturated fat, carbohydrate and dietary fiber) and micronutrient intake (sodium, calcium and cholesterol) were assessed by means of a 24-hour food recall that was applied to 540 individuals. These subjects were interviewed in person by a dietitian (who was also the fieldwork coordinator) or by trained undergraduate nutrition students under the supervision of the same dietitian. A second 24-hour food recall was applied on non-consecutive days, within a one-week interval, to 30% of the sample (n = 161). This second measurement was made via telephone by the same evaluator, and only if the participant agreed to be contacted. It enabled correction of the within-individual variability in food intake.^12^

Out of the 540 participants, 32 were excluded from the present analysis because they reported a total energy intake below the third percentile (768.4 kcal) or above the 97^th^ percentile (5,680.7 kcal), and therefore were considered outliers (possible dietary intake underreporting or overreporting, i.e. the intakes were considered unlikely to be correct^13^). Another participant was excluded because of an incomplete questionnaire. Data on 507 individuals were then analyzed. Among these, 31% gave responses for a second 24-hour food recall.

Detailed descriptions of food types and serving sizes consumed the day before the interview were obtained using rigorous standardization, and these data were inputted into the NutWin software (Departamento de Informática em Saúde/Escola Paulista de Medicina [DIS-EPM], São Paulo, Brazil). Standardizations were based on Brazilian Dietary Guidelines.^14^^,^^15^

Foods that were not found in the NutWin software datasets were included by using the following nutrient databases hierarchically: TACO (Tabela Brasileira de Composição de Alimentos),^16^ TBCA-USP (Tabela Brasileira de Composição de Alimentos-USP)^17^ and NNDSR-USDA (National Nutrient Database for Standard Reference, United States Department of Agriculture).^18^ For any processed foods for which nutritional information was not available, data were obtained from the nutritional fact labels, available on the food companies’ websites.

Sociodemographic data were collected using a standardized questionnaire and included age, gender and education level. Clinical data, which included CD4 cell count (cells/mm^3^) analyzed by means of flow cytometry (FACSCalibur, BD) and viral load (copies/ml) measured using branched deoxyribonucleic acid (DNA) assay (Versant440, Siemens), were obtained from the participants’ medical files. The maximum time interval between the interview date and the date of obtaining the clinical data was approximately six months. The dates of the first positive HIV diagnosis and onset of HAART use were also obtained from the participants’ medical files. The viral load was analyzed as a dichotomous variable (detectable or undetectable), taking the threshold to be 50 copies/ml. The sample sizes for the analyses of CD4 cell count and viral load were 501 and 473, respectively, due to missing values.

Body mass index (BMI) was used to characterize the nutritional status of the population, and it was estimated from self-reported measurements of height and weight. This methodology has been validated and published elsewhere.^19^

### Statistical analysis

Initially, descriptive analysis was performed. Food intake was presented as absolute measurements and density (total intake of each nutrient per 1,000 kcal consumed). Means were compared using Student’s t test, and categorical variables were analyzed using Pearson’s chi-square test. The significance level accepted was 5%. The statistical analyses were performed using SPSS (Statistical Package for the Social Sciences), version 17.0 (SPSS Inc., Chicago, IL, USA).

The prevalence of inadequate food intake was estimated by comparing the population’s intake with the dietary reference intake (DRI) reference values for carbohydrates, lipids, proteins, dietary fiber, calcium and sodium.^20^ For micronutrients, the estimated average requirement (EAR) was the recommended standard. Since for dietary fiber intake there are still not enough studies to allow the establishment of EAR values, fiber intake was assessed using adequate intake (AI) values. Although this did not allow the percentage of inadequate intake to be estimated, it allowed us to conclude whether the intake was probably adequate.

Sodium intake was evaluated based on its upper limit (UL) of tolerable intake. Although its EAR is available, the general population’s intake is usually much higher than what is recommended,^21^ and easily surpasses this threshold. Therefore, it is more realistic to ascertain its appropriateness in relation to the maximum tolerated level. For saturated fat and cholesterol, the reference values recommended by the World Health Organization^22^ (which are also the recommendations of the Dietary Guidelines for the Brazilian Population^10^) were used, since no established DRI values are yet available for either of these nutrients (Table 1).

**Table 1.** Food intake recommendations

Nutrient		Recommendation
Carbohydrate		45% to 65% of the TEV^*†^
Protein		10% to 35% of the TEV^†^
Total fat		20% to 35% of the TEV^†^
Saturated fat		< 10% of the TEV^‡^
Dietary fiber	Females 19 to 50 years old	25 g^†a^
	Females 51 to 59 years old	21 g^†a^
	Males 19 to 50 years old	38 g^†a^
	Males 51 to 59 years old	30 g^†a^
Cholesterol		< 300 mg^‡^
Calcium	Females 19 to 50 years old	800 mg^†b^
	Females 51 to 59 years old	1,000 mg^†b^
	Males 19 to 59 years old	800 mg^†b^
Sodium		2,300 mg^†c^

^*^TEV = total energy value; ^†^Dietary reference intakes;^20 ‡^World Health Organization;^4^ ªAdequate intake; ^b^Estimated average requirement; ^c^Tolerable upper intake limit.

The Iowa State University (ISU) method, developed by Nusser et al.,^23^ was used to adjust the between and within-individual distribution of the variability. This procedure requires at least a second 24-hour food recall to be conducted on a subsample of the study population, and creates descriptive measurements of intake and the between and within-individual variance components. Variance component estimates and models for estimating the distribution of the usual intake were developed on the PC-side software, version 1.0.^23^

The adequacy of carbohydrate, protein and fat intake (equivalent to proportional ranges from the total energy intake) was estimated as the sum of percentages above and below the range of nutrient recommendations. For the other nutrients, the percentages relating to the intake below the recommended level (in the case of dietary fiber and calcium) or above the recommended level (in the case of saturated fat, cholesterol and sodium) were used.

All the nutrient levels in the study population were homogeneous regarding gender and age, except for calcium and dietary fiber, for which the results were presented stratified by age and gender (Table 1).

## RESULTS

A total of 507 patients were evaluated, with a mean age of 41.7 years (standard deviation, SD = 7.8 years), of whom 57.8% were men. The majority reported that they had had eight years of education or less (54.2%), and 60.3% of the sample presented healthy weight (BMI between 18.5 and 24.9 kg/m^2^). Only 5.4% were underweight, whereas 34.3% were overweight or obese. The mean time since the first HIV-positive diagnosis was 6.6 years (SD = 4.1 years), and the mean time since the onset of HAART use was 5.1 years (SD = 3.3) (Table 2).

**Table 2.** Characterization of the studied population according to gender (São Paulo, 2008)

		Total	Male	Female
		n (%)*	n (%)	n (%)
Age (years)	20 to 29	33 (6.5)	18 (6.1)	15 (7.0)
	30 to 39	182 (35.9)	96 (32.8)	86 (40.2)
	40 to 49	210 (41.4)	124 (42.3)	86 (40.2)
	> 50	82 (16.2)	55 (18.8)	27 (12.6)
Schooling (years)	? 8	274 (54.2)	148 (50.7)	126 (58.9)^†^
	8 to 11	173 (34.2)	99 (33.9)	74 (34.6)
	> 11	59 (11.7)	45 (15.4)	14 (6.5)
TCD4+ cells (cells/mm^3^)^a^	< 200	86 (17.2)	49 (16.8)	37 (17.6)
	200 to 499	222 (44.3)	138 (47.4)	84 (40.0)
	³ 500	193 (38.5)	104 (35.7)	89 (42.4)
Viral load (copies/ml)^b^	Undetectable	137 (29.0)	76 (26.9)	61 (32.1)
	Detectable	336 (71.0)	207 (73.1)	129 (67.9)
BMI (kg/m^2^)	< 18.5	26 (5.4)	7 (2.5)	19 (9.8)^†^
	18.5 to 24.99	288 (60.3)	178 (62.5)	110 (57.0)
	25 to 29.99	130 (27.2)	87 (30.5)	43 (22.3)
	³ 30	34 (7.1)	13 (4.5)	21 (10.9)
Disclosure of diagnosis				
Family		392 (77.6)	216 (74.0)	176 (82.6)^†^
Partner		240 (89.6)	140 (93.3)	100 (84.7)^†^
Adherence to HAART	< 95%	241 (48.3)	146 (50.7)	95 (45.0)
	³ 95%	258 (51.7)	142 (49.3)	116 (55.0)
Time since HIV diagnosis (years)^‡^		6.61 (4.08)	6.35 (4.14)	6.97 (3.97)
Duration of HAART (years)^‡^		5.11 (3.30)	5.10 (3.27)	5.12 (3.35)

^*^Except where indicated otherwise; ^†^P < 0.05; ^‡^Mean (standard deviation); BMI = body mass index; HAART = highly active antiretroviral therapy; ^a^sample size = 501; ^b^sample size = 473.

After correcting for both the between and the within-individual variability, the mean habitual energy intake of the population was 2,333 kcal (SD = 377 kcal) (Table 3). The mean sodium and calcium intake were respectively 4,084.5 mg (SD = 472.4 mg) and 517.3 mg (SD = 122.4 mg). The macronutrient intake differed significantly according to gender (P < 0.01). Men had higher intakes of carbohydrates (340.3 g, SD = 84.8 g versus 284.6 g, SD = 76.2 g), protein (108.9 g, SD = 17.9 g versus 94.9 g, SD = 16.9 g), total fat (74.8 g, SD = 12.9 g versus 67.5 g, SD = 12.3 g) and saturated fat (23.0 g, SD = 4.5 g versus 21.2 g, SD = 4.7 g).

**Table 3.** Food intake adjusted according to the within-individual variability, among adults living with HIV (São Paulo, 2008)

	Total	Male	Female
	Mean (SD)	Mean (SD)	Mean (SD)
Energy (kcal)	2332.92 (377.73)	2334.21 (365.01)	2331.15 (395.33)
Carbohydrate (g)	316.75 (85.72)	340.27 (84.75)	284.55 (76.20)^*^
Protein (g)	102.97 (18.78)	108.86 (17.88)	94.89 (16.92)^*^
Total fat (g)	71.68 (13.14)	74.76 (12.90)	67.46 (12.30)^*^
Saturated fat (g)	22.25 (4.66)	23.04 (4.45)	21.18 (4.74)^*^
Cholesterol (mg)	316.73 (69.10)	316.55 (64.41)	316.97 (75.20)
Dietary fiber (g)	25.39 (6.86)	28.78 (6.53)	20.74 (3.98)^*^
Sodium (mg)	4084.54 (472.40)	4084.07 (478.58)	4085.18 (464.92)
Calcium (mg)	517.34 (122.37)	515.56 (117.99)	519.76 (128.37)

^*^P < 0.05; SD = standard deviation.

The mean intake of saturated fat was 9.6 g/1,000 kcal (SD = 1.7 g) (Table 4). Sodium and calcium presented mean densities of 1775.0 mg (SD = 218.4 mg) and 223.1 mg (SD = 45.5 mg) per 1,000 kcal, respectively.

**Table 4.** Food intake density adjusted according to the within-individual variability, among adults living with HIV (São Paulo, 2008)

	Mean (SD)
Carbohydrate (g/1000 kcal)	134.51 (21.76)
Protein (g/1000 kcal)	44.34 (5.72)
Total fat (g/1000 kcal)	30.84 (3.64)
Saturated fat (g/1000 kcal)	9.60 (1.69)
Cholesterol (mg/1000 kcal)	136.40 (23.77)
Dietary fiber (g/1000 kcal)	10.97 (2.68)
Sodium (mg/1000 kcal)	1774.99 (218.44)
Calcium (mg/1000 kcal)	223.05 (45.48)

SD = standard deviation.

The macronutrient intake was within the recommended range for the majority of the population, except for dietary fiber, which was low for 65% of the sample (Table 5).

**Table 5.** Prevalence of food intake above or below the recommendations among adults living with HIV (São Paulo, 2008)

Nutrient		Intake above or below the recommendations
		%
Carbohydrate (% TEV)		6.40^*^
Protein (% TEV)		0.00^*^
Total fat (% TEV)		7.18^*^
Saturated fat (% TEV)		24.63ª
Cholesterol (mg)		53.69ª
Sodium (mg)		99.28^b^
Calcium (mg)	19 to 50-year-old females (n = 191)	93.92^*^
	51 to 59-year-old females (n = 23)	100.00^*^
	19 to 59-year-old males (n = 293)	86.05^*^
Dietary fiber (g)	19 to 50-year-old females (n = 191)	73.97^†^
	51 to 59-year-old females (n = 23)	67.24^†^
	19 to 50-year-old males (n = 248)	81.83^†^
	51 to 59-year-old males (n = 45)	69.83^†^

TEV = total energy value; ^*^Inadequate intake; ^†^Below the recommended adequate intake;

ªAbove the levels recommended by the World Health Organization; ^b^Above the recommended tolerable upper intake limit.

Using the UL of 2,300 mg to evaluate the proportion of individuals with excessive sodium intake, it was found that 99% had an intake above the maximum tolerable level. For calcium, among women between 19 and 50 years old, 94% had an intake below the recommended level (800 mg). Among women over 50 years old, none of them reached the recommendation (1,000 mg). Among men, 86% had inadequate intake.

## DISCUSSION

This study examined the prevalence of inadequate food consumption among adults living with HIV in São Paulo, Brazil. The analysis revealed that there was high prevalence of inappropriate levels of saturated fat, cholesterol, calcium, dietary fiber and sodium.

The prevalence of intake below or above the recommendations was lower than 8% for carbohydrates, protein and total fat, despite the excessive intake of some nutrients and insufficient intake of others. Different results were found by Hendricks et al.^24^ in a study among American PLHIV, in which the intake of total fat was higher than the recommended level for more than half of the population.

Excessive saturated fat and cholesterol intake is associated with some of the side effects from antiretroviral therapy and may result in a disturbing propensity towards deposition of visceral fat and increased risk of cardiovascular diseases.^7^ On the other hand, the levels of dietary fiber, which is known to be a protective factor against cardiovascular diseases, and helps to control insulin resistance and dyslipidemia,^25^^,^^26^ were inadequate in 65% of the population. This proportion reached 84% among men between 19 and 50 years. Similar results were observed in an American study on PLHIV, in which the recommended amount of fiber intake per day (20 g/day) was lower than the values used in the present study. Even so, more than half of the individuals presented lower intake of fiber, especially among obese women (94%).^24^

These results suggest that this population may have a low-quality diet, characterized by a low intake of whole grains, fruits and vegetables, which are good sources of fiber, thus corroborating previous findings from a sample of adults living with HIV in the city of São Paulo.^27^

The majority of the population had a low calcium intake, which has been associated with osteoporosis, hypertension, obesity and cervical cancer.^28^ A similar result was observed by Leite and Sampaio.^29^

However, the sodium intake was high for virtually the entire population. This result expresses a potential risk of adverse health effects, such as gastric cancer, hypertension, osteoporosis, stroke and cardiovascular diseases, which are closely connected with other risks arising from inadequate intake (excessive or insufficient) of saturated fat, fiber and cholesterol, for example.^30^^,^^31^^,^^32^

There is still a scarcity in the literature of studies estimating sodium intake in the population, and even more so in relation to PLHIV, in part because of the difficulty in measuring its consumption. Another limitation is the lack of accurate measurement of the sodium content of foods consumed by individuals in most food composition databases. A study using data from the Brazilian Family Budget Survey (Pesquisa de Orçamentos Familiares, POF) from 2002-2003 showed that the average amount of sodium available for consumption was 4.5 g per person per day, and was even higher in rural households than in urban ones.^33^

The prevalence of overweight or obesity was high in this population (34%), especially among women, and this corroborates what has been observed in the Brazilian population in general. Between 1975 and 2003, the prevalence of obesity in adults increased from 2.7% in men and 7.4% in women to 8.8% and 13.0% respectively.^34^ In 2009, it was seen that obesity had continued to increase: its prevalence was 12.5% in men and 16.9% in women.^35^ Data from telephone-based surveillance of risk and protective factors for chronic diseases (Vigilância de Fatores de Risco e Proteção para Doenças Crônicas por Inquérito Telefônico, VIGITEL) collected in 27 Brazilian state capitals in 2010 showed that the prevalence of overweight (BMI ³ 25 kg/m^2^) was 48.5% (95% confidence interval, CI: 45.2-51.8) and obesity (BMI ³ 30 kg/m^2^), 15.0% (95% CI 12.8-17.2) in the city of São Paulo.^36^

The issue of the increasing prevalence of overweight in HIV-infected populations, also shown by Jaime et al.,^37^ corroborates the trend of the nutritional transition process observed in the Brazilian population, from a malnutrition profile to a profile anchored in overweight.^38^ These results derive directly from the choices that people make relating to their own food intake and physical activity.

This study is subject to a few limitations. One is in relation to the sampling method chosen, which was a convenience sample, although improved through a consecutive cumulative approach. Another limitation relates to the method chosen for measuring food intake: 24-hour food recalls depend on the interviewee’s memory, and refer to the current diet, not the habitual diet. On the other hand, it was possible to reduce the effects of both the between and the within-individual variances, thereby making it possible to improve the measurement accuracy. Moreover, this methodology has been shown to be adequate.^12^^,^^39^

Thirdly, we chose to apply the second 24-hour food recall to only 30% of the sample via telephone. Although the accuracy of this approach might be limited in a group of low education level, it has been shown that interviews conducted by telephone to collect dietary data have good reproducibility, and that respondents of different ages and education levels understand the questions and have no difficulty in answering them.^40^

As obesity among HIV-infected populations becomes an important issue, the prevalence of metabolic syndrome may increase, thus requiring new clinical care for these specific conditions in this population.^24^ In the general population, there is extensive evidence of beneficial effects derived from maintaining normal levels of plasma lipoproteins, with weight control and blood pressure control, in order to reduce the risk of cardiovascular diseases and increase the quality of life.^41^ Thus, diet has an important role in modifying these risk factors among people living with HIV.

## CONCLUSIONS

Most of the population reported an adequate intake of macronutrients, except for saturated fat and dietary fiber. Moreover, virtually the entire sample had excessive sodium intake and low calcium intake. Actions to promote healthy eating should be included in designing public policies, with the aim of improving the quality of life of PLHIV.
